# MicroRNAs as Regulators of Neuroinflammation in Major Depressive Disorder

**DOI:** 10.1155/da/9984291

**Published:** 2025-12-11

**Authors:** Qirui Li, Yuyan Ling, Ling Gu, Lei Li, Yutong Liu, Yuanyuan Ma, Ruiting Ma, Meijuan Chen

**Affiliations:** ^1^ School of Medicine, Nanjing University of Chinese Medicine, Nanjing, 210023, Jiangsu Province, China, njucm.edu.cn; ^2^ School of Chinese Medicine, Nanjing University of Chinese Medicine, Nanjing, 210023, Jiangsu Province, China, njucm.edu.cn; ^3^ Mongolian Medicine Department, The Affiliated Hospital of Inner Mongolia Medical University, Hohhot, 010050, Inner Mongolia Autonomous Region, China, nmgfy.com; ^4^ Medical Laboratory Department, Inner Mongolia Autonomous Region Mental Health Center, Hohhot, 010010, Inner Mongolia Autonomous Region, China

## Abstract

Major depressive disorder (MDD) is a globally prevalent mental health condition with a complex pathogenesis and substantial disease burden. However, due to incomplete mechanistic understanding, existing therapeutic strategies frequently yield suboptimal outcomes. This review synthesizes evidence establishing neuroinflammation as a central pathogenic mechanism of MDD, involving elevated proinflammatory cytokines, microglial M1 polarization, blood–brain barrier (BBB) disruption, hypothalamic–pituitary–adrenal (HPA) axis dysregulation, and impaired neuroplasticity. Micro ribonucleic acid (miRNA) are identified as master molecular regulators bridging neuroinflammation and MDD pathology with details of how specific dysregulated miRNAs orchestrate MDD processes by targeting key inflammatory pathways, directing microglial polarization states, mediating intercellular communication via exosomes, and modulating BBB integrity. Crucially, these miRNAs may serve as novel diagnostic biomarkers and therapeutic targets for MDD. Building on this, we explore the potential of natural compounds as innovative miRNA‐targeting therapeutics that can ameliorate neuroinflammation and restore neuroplasticity. Current challenges relating to clinical translation are discussed, including discordance between peripheral and brain miRNA profiles, species‐specific miRNA functional variations, limited biomarker specificity across psychiatric disorders, the absence of standardized clinical reference ranges, and the need for more effective delivery systems. Overall, this review positions miRNA‐mediated neuroinflammation regulation as a transformative frontier for MDD pathogenesis research and targeted treatment.

## 1. Introduction

Major depressive disorders (MDDs), which is characterized by ≥2 weeks of persistent depressed mood causing significant functional impairment, affect ~280 million people globally (lifetime prevalence: males 4%, females 6%) [1]. MDD is associated with an increased risk of co‐occurring health conditions, including cardiovascular/cerebrovascular disease, respiratory conditions, diabetes, infections, and malignancies, which can worsen their prognosis. Overall, MDD is a substantial contributor to mortality, disability, unemployment, and socioeconomic burden worldwide [2–4]. Existing treatments for MDD, whether pharmacological, psychological, or biophysical, often fail to prevent replace, reflecting MDD’s multifactorial etiology (genetic predisposition, environmental stressors) and the lack of definitive molecular targets [5].

### 1.1. Pathogenic Mechanisms of MDD

The cornerstone etiological model of MDD is the monoaminergic hypothesis, which posits that deficiencies in the neurotransmission of norepinephrine, serotonin, and dopamine disrupt synaptic signaling and neural circuit functionality [6]. Serotonin, in particular, has served as the primary therapeutic target and biomarker of MDD [7]. However, the frequent inefficacy of neurotransmitter‐precursor antidepressants [8, 9] and their characteristic delayed onset [10] challenge this paradigm, necessitating more sophisticated pathophysiological frameworks.

Emerging evidence supports an alternative mechanism termed the receptor dysplasticity hypothesis, which proposes that chronic synaptic neurotransmitter depletion induces compensatory postsynaptic receptor overexpression and hypersensitivity [11]. Consequently, therapeutic efficacy may require concurrent normalization of neurotransmitter availability and receptor density/function. Supporting this concept: Patients exhibit decreased functional connectivity correlating with symptom severity and serotonin 1A receptor (5‐HT1AR) distribution [12]; That pharmacological disruption of 5‐HT1AR/orexin receptor heterodimers has antidepressant effects [13], and that MDD risk factors (e.g., childhood adversity) are associated with epigenetic 5‐HT1AR hypermethylation impacting receptor expression [14], a process involving reversible chemical modifications and noncoding ribonucleic acid (RNA)‐mediated regulation of gene expression patterns without alteration of the deoxyribonucleic acid (DNA) sequence itself.

Neuroendocrine dysregulation is another prominent feature of MDD pathogenesis. The hypothalamic–pituitary–adrenal (HPA) axis is the core neuroendocrine pathway by which the organism responds to internal and external stressors. Its overactivation can present as dexamethasone‐resistant hypercortisolaemia and hypothalamic dysfunction [15], with prolonged cortisol exposure potentially inducing hippocampal atrophy via neurotoxic mechanisms [11, 16]. This neurostructural compromise may contribute to pervasive cognitive impairment observed in patients with MDD [17, 18]. Associations have also been reported between MDD and HPA axis dysfunction, with Mendelian randomization suggesting bidirectional causality with hypothyroidism [19]. Clinical studies also indicate that comorbid thyroid‐stimulating hormone abnormalities are correlated with heightened depression severity, anxiety, and suicide risk [20], although causality with hyperthyroidism remains debated [19], and thyroid hormone levels show an inconsistent risk association [21].

### 1.2. MDD and Neuroinflammation

Neuroinflammation describes inflammatory responses occurring within the central nervous system (CNS). This process is primarily mediated by the activation of neuroimmune cells, such as microglia and astrocytes, and accompanied by the release of proinflammatory cytokines (e.g., interleukin (IL)‐1*β*, IL‐6, tumor necrosis factor (TNF)‐*α*), chemokines, and reactive oxygen species (ROS). Converging evidence implicates neuroinflammation as a critical pathogenic mechanism of MDD. For example, patients with MDD demonstrate elevation of peripheral and central inflammatory markers [22–24], first‐line antidepressants reduce proinflammatory cytokines (e.g., IL‐6, TNF‐*α*) [25], inflammatory comorbidities (e.g., arthritis, hepatitis), increase susceptibility to MDD, and anti‐inflammatory agents are reported to have therapeutic potential [5, 25].

Micro RNAs (miRNAs) are evolutionarily conserved, noncoding single‐stranded RNA molecules, typically 18–25 nucleotides long, that play critical roles in various biological processes. These processes include those relating to neuroinflammation, with increasing evidence that miRNAs are master modulators of neuroinflammatory pathways in MDD [11, 26]. For example, dysregulated miRNA expression profiles in blood and postmortem brain tissue samples from patients with MDD [27] have been shown to functionally contribute to disease progression. Another study reported that empagliflozin, an antidepressant, ameliorates depressive phenotypes by downregulating miR‐134, suppressing IL‐1*β*/IL‐6/TNF‐*α* production, and restoring brain‐derived neurotrophic factor (BDNF) signaling [28]. Critically, specific miRNAs have been shown to exert bidirectional control over neuroinflammatory cascades (promoting resilience or vulnerability) via intricate regulatory networks [29–32].

Given growing interest in the relationship between inflammation and MDD, this review synthesizes current understanding of MDD pathogenesis with emphasis on neuroimmune mechanisms and miRNA‐mediated regulation. We also identify neuroinflammation‐modulating natural compounds that may confer antidepressant effects via selective miRNA targeting, proposing a novel therapeutic strategy for MDD.

## 2. Main Neuroinflammatory Mechanisms in MDD

### 2.1. Major Proinflammatory Factors

There are numerous reports of significantly elevated levels of various inflammatory mediators, including TNF‐*α*, C‐reactive protein (CRP), IL‐1*β*, IL‐6, IL‐12, and IL‐18, in serum and CNS tissues samples from patients with MDD and relevant animal models [23, 24, 28, 33]. These cytokines and acute‐phase reactants have been shown to elicit depressive‐like symptomatology in preclinical systems [23, 24, 34, 35] and disrupt key neurochemical pathways through diverse mechanisms, contributing to depressive symptom exacerbation. Beurel et al. [36] highlight differential associations between specific inflammatory biomarkers and depression subtypes, finding that elevated IL‐6 levels predicted chronicity while increased TNF‐*α* and CRP levels predicted inflammation‐associated treatment‐resistant depression.

### 2.2. Primary Mechanisms Through Which Inflammatory Factors Contribute to Depression

**Figure 1:**
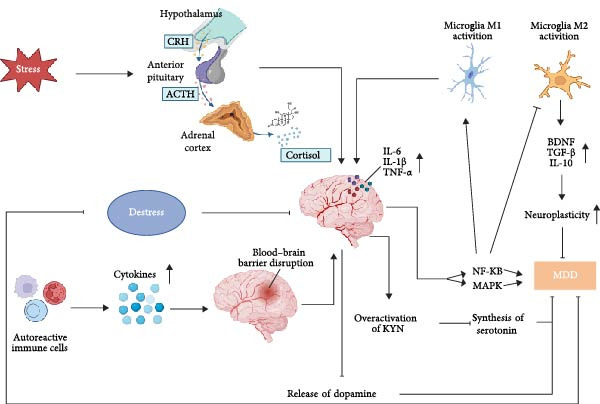
Possible mechanisms through which neuroinflammation induces MDD. Figure created using BioRender (https://www.biorender.com). *Note:* Neuroinflammation contributes to depression primarily through the following mechanisms: (1) Proinflammatory cytokine release compromises BBB integrity, facilitating neuroinflammatory exacerbation [37]; (2) MAPK and NF‐κB pathway activation induces dysregulation of depression‐associated gene expression [38–40]; (3) Kynurenine pathway hyperactivation suppresses serotonin synthesis via tryptophan metabolism diversion [41, 42]; (4) dopaminergic neurotransmission impairment through reduced presynaptic dopamine release [43]; (5) HPA axis dysregulation via impaired glucocorticoid negative feedback, sustaining chronic hypercortisolemia and neuroinflammation [5, 44]; (6) microglial polarization imbalance: M1 polarization promotes neuroinflammation through proinflammatory cytokine secretion [34, 45], whereas M2 polarization exerts neuroprotective effects through anti‐inflammatory mediator release [46–48]. ACTH, adrenocorticotropic hormone; CRH, corticotropin‐releasing hormone; IL, interleukin; KYN, kynurenine; MAPKs, mitogen‐activated protein kinases; MDD, major depressive disorder; NF‐κB, nuclear factor kappa‐B; TNF‐*α*, tumor necrosis factor‐alpha.

Neuroinflammation is thought to contribute to MDD pathogenesis through multiple mechanisms (Figure 1): compromising effects on the structural and functional integrity of the blood–brain barrier (BBB), dysregulation of mitogen‐activated protein kinases (MAPKs), and nuclear factor kappa‐B (NF‐κB) signaling pathways, inhibition of serotonin synthesis and dopamine release, disruption of HPA axis homeostasis, and alteration of microglial polarization states.

BBB disruption, which involves both structural compromise and functional impairment, is a significant neuropathological feature in depression and other CNS disorders [49]. Under physiological conditions, the BBB maintains cerebral microenvironmental homeostasis by restricting transendothelial migration of immune cells and diffusion of inflammatory mediators [49, 50]. However, proinflammatory cytokines can initiate neuroinflammatory cascades within the CNS by disrupting BBB integrity [37]. Utilizing dynamic contrast‐enhanced magnetic resonance imaging, Shang et al. [51] observed significantly higher mean volume transfer constant values (indicative of BBB permeability) in the olfactory region, caudate nucleus, and thalamus of patients with MDD compared to healthy controls. Moreover, they found that patients with MDD receiving treatment exhibited lower values in the orbital frontal lobe, anterior cingulate gyrus, putamen, and thalamus relative to untreated counterparts. They also reported that these values positively correlated with Hamilton Depression Rating Scale and Hamilton Anxiety Scale scores across multiple brain regions, supporting a relationship between BBB permeability abnormalities and greater MDD symptoms. Fronza et al. [52] reported that the antidepressant effects of acetylcholinesterase, glycogen synthase kinase 3*β* (GSK3*β*), and *β*‐secretase in mice correlate with normalization of BBB permeability. Claudin‐5, the principal BBB tight junction protein, has also been shown to demonstrate consistent downregulation across diverse MDD models [53]. A study by Sun et al. [54] suggested that targeted upregulation of Claudin‐5 expression could attenuate stress‐induced translocation of TNF‐*α* into the hippocampus, reverse BBB damage, and ameliorate depressive behaviors in chronic unpredictable mild stress (CUMS) mice. However, whether BBB impairment is a primary etiological factor or a secondary consequence in human depression remains unclear.

Intracerebral activation of inflammatory mediators can engage various signaling pathways within neural cells that modulate the expression of depression‐associated genes [38–40]. One such pathway is NF‐κB, which serves as a master regulator of neuroinflammatory signaling by facilitating the release of inflammatory cytokines (e.g., TNF‐*α*, IL‐6) following activation via the Toll‐like receptor 4 (TLR4)/myeloid differentiation primary response gene 88 (MyD88) complex formation [55]. Pharmacological inhibition of MyD88 has been shown to reduce ROS production by suppressing the MAPK pathway, ultimately attenuating inflammation [56], while disruption of TNF receptor‐associated factor 6 (TRAF6)/NF‐κB signaling modulates inducible nitric oxide synthase expression and promotes microglial M2 polarization via the MAPK/protein kinase B (Akt) axis [57, 58]. Alzarea et al. [59] reported that lipopolysaccharide (LPS)‐induced neuroinflammation and depression in mice are mitigated by peroxisome proliferator‐activated receptor *α* (PPAR‐*α*) agonism, with PPAR‐*α* suppressing NF‐κB expression and exerting antidepressant and procognitive effects that can be reversed by PPAR‐*α* antagonism. A clinical study by Savitz et al. [60] found that cytomegalovirus infection exacerbates depressive symptoms in patients with MDD by enhancing NF‐κB activity, with reductions in viral antibody titers or cluster of differentiation (CD) 8^+^ T cell activity correlating with reduced depressive symptomatology and NF‐κB signaling. Moreover, the efficacy of several antidepressants has been shown to mechanistically correlate with NF‐κB downregulation [39, 52, 59, 61]. The prodepressant effects of NF‐κB are thought to relate to its downstream high mobility group box 1/signal transducer and activator of transcription 3/p65 pathway, as supported by Zheng et al. [61] and Xu et al. [62]. Joo et al. [39] reported a relationship between elevated NF‐κB levels and reduced BDNF mRNA expression in a rodent depression model. The BDNF/tropomyosin receptor kinase B (TrkB) signaling axis is another core regulator of neuroplasticity that shows evidence of dysregulation in depression [63]. Consequently, activation of BDNF/TrkB signaling is a common pharmacological endpoint of conventional antidepressants [64]. The binding of BDNF to TrkB triggers receptor autophosphorylation and induces BDNF gene transcription, serving as an autocrine loop critical for BDNF‐mediated neuroprotection [63]. Significantly elevated BDNF promoter methylation has been observed in postmortem brain tissue samples from suicidal patients with MDD compared to controls, suggesting that epigenetic suppression of the BDNF/TrkB pathway contributes to MDD [65]. Karad et al. [40] identified neuroprotective natural compounds capable of upregulating BDNF expression via MAPK/NF‐κB modulation, enhancing synaptic plasticity, and attenuating neuroinflammation, ultimately improving affective and cognitive function. Li et al. [66] reported molecular convergence onto MAPK signaling across diverse depression‐inducing stress models, such as CUMS, chronic social defeat stress (CSDS), chronic restraint stress (CRS), and learned helplessness. Kwatra et al. [38] reported concurrent elevation of inflammatory mediators and MAPK signaling intensity within the hippocampus and frontal cortex of CRS mice, which was exacerbated by LPS challenge. Collectively, these findings implicate cytokine‐mediated dysregulation of MAPK/NF‐κB/BDNF networks in depression pathophysiology..

In addition to their ability to activate signaling pathways directly, proinflammatory cytokines can result in overactivation of the kynurenine pathway to decrease synthesis of the mood‐regulatory neurotransmitter serotonin. Okamoto et al. [67] found that patients with MDD who also had comorbid type 2 diabetes mellitus and elevated systemic inflammation exhibit diminished antidepressant treatment response. The finding of significantly altered kynurenine, serotonin, and TNF‐*α* levels in this cohort suggest that adjunctive anti‐inflammatory therapy may enhance treatment efficacy. Indoleamine 2,3‐dioxygenase and kynurenine monooxygenase are both kynurenine pathway enzymes capable of degrading the serotonin precursor tryptophan. The downstream metabolites generated through the degradation of tryptophan by these two enzymes, including quinolinic acid, which have been shown to induce oxidative stress, thereby generating ROS (e.g., hydrogen peroxide, hydroxyl radicals) that can propagate cycles of neuroinflammation and neuronal damage [5, 41, 42]. Based on such findings, kynurenine metabolites have been proposed as candidate biomarkers of MDD, although consensus of these proteomic signatures require further validation [68].

Neuroinflammation can also contribute to depression by attenuating neural activity and dopamine release within reward‐related circuits, manifesting as the clinical symptoms anhedonia and reduced motivation. A clinical study stratifying patients with MDD by CRP levels reported enhanced corticostriatal circuit responsiveness to the dopamine precursor levodopa specifically among those with CRP levels > 2 mg/L [69]. Similarly, Burrows et al. [70] reported diminished striatal activation during reward processing in patients with MDD and serum CRP > 3 mg/L versus those with lower levels. Indicating a potential mechanism, Singh et al. [43] reported greater infliximab‐induced anhedonia reduction compared to placebo, specifically among patients with depression and CRP levels > 3 mg/L, concomitant with increased dopamine release. Taken together, these observations implicate CRP‐mediated inhibition of dopaminergic neurotransmission in depression pathogenesis, with further investigation of the underlying mechanisms warranted.

Neuroinflammation processes engage in complex bidirectional crosstalk with HPA axis regulation. Stress induction activates the sympathetic nervous system, inducing the release of catecholamine (adrenaline and noradrenaline) that potentiate the secretion of proinflammatory cytokines (e.g., TNF‐*α*, IL‐6) and enhance macrophage phagocytosis. Conversely, stress resolution activates the parasympathetic nervous system and results in the release of acetylcholine, which suppresses the production of proinflammatory cytokines (TNF‐*α*, IL‐1*β*, IL‐6, and IL‐18). Impaired sympathetic nervous system activity in MDD manifests as reduced acetylcholine availability, enabling sustained accumulation of proinflammatory mediator [5]. Within this chronic inflammatory milieu, cortisol loses its physiological efficacy, impairing negative feedback of the HPA axis. This impairment culminates in resistance of glucocorticoid receptors within immune cells, limiting peripheral anti‐inflammatory actions. Persistently elevated inflammation further attenuates glucocorticoid sensitivity, reducing corticotropin‐releasing hormone production suppression and exacerbating stress. This cascade results in decreased serotonin availability and dysregulated glutamatergic neurotransmission, suppressing neurogenesis and promoting cytopathology within mood‐regulatory circuits [44].

Microglia, which are the primary immune cells within the CNS, orchestrate astrocyte activation, synaptic modulation, and neuronal support [71]. Zhang et al. [72] suggested that CUMS mice also show hippocampal upregulation of circular RNA (circRNA) protein tyrosine phosphatase 4A2, with hippocampal ablation attenuating both depressive‐like phenotypes and microglial activation. A study that administered small interfering RNA (siRNA) targeting circRNA activating transcription factor 7 interacting protein and PPAR‐*α* agonists into the hippocampus of LPS‐treated mice demonstrated reduced microglial activation and proinflammatory cytokine expression (TNF‐*α*, IL‐1*β*, and IL‐6) [59, 73]. Microglial polarization, which refers to the dynamic process of functional phenotype switching regulated by microenvironmental signals, may underlie the association with inflammation and MDD. This plasticity enables microglia to differentiate into two primary phenotypes: proinflammatory (M1) and anti‐inflammatory (M2) [74]. Preclinical evidence indicates M1‐polarized microglia exacerbate depressive symptoms in CUMS models via excessive proinflammatory cytokine release (TNF‐*α*, IL‐1*β*, and IL‐6) [34, 45]. In contrast, M2‐polarized microglia have been shown to promote tissue repair, clear debris, increase neurotrophic factor secretion, and reduce proinflammatory cytokine burden, ultimately attenuating depressive‐like behaviors in CUMS and CSDS mice [46–48]. The anti‐inflammatory cytokine milieu associated with M2 polarization, including transforming growth factor‐beta (TGF‐*β*) and IL‐10, thus has significant antidepressant effects. Zhao et al. [48] identified M2 microglial activation as essential for the antidepressant efficacy of *β*‐glucan, finding that microglial depletion abolished both M2 polarization and behavioral improvement. Similarly, Wang et al. [75] established that M2 microglia attenuate calreticulin membrane translocation and rectify aberrant phagocytosis and neuroinflammation, conferring antidepressant effects. A clinical study by Tifner et al. [76] documented significantly reduced CD4^+^IL‐10^+^ T‐helper cell frequencies in peripheral blood samples from patients with MDD, which inversely correlated with depression chronicity and recurrence. Xu et al. [77] reported that a TGF‐*β* receptor inhibitor blocks the antidepressant effects of arketamine. Mechanistically, IL‐10 enhances synaptic plasticity via BDNF/TrkB signaling and restores phosphatidylinositol 3‐kinase (PI3K) expression/Akt signaling to suppress GSK3*β* activity, ameliorating depressive‐like behaviors in mice [78, 79]. TGF‐*β* promotes M2 polarization and facilitates vagus nerve‐mediated spleen‐brain axis, impacting neuroprotection and antidepressant efficacy [80, 81]. These collective findings underscore the pivotal antidepressant role of M2 microglia and associated anti‐inflammatory cytokines. Given evidence of NF‐κB‐mediated modulation of BDNF signaling and microglial polarization, we hypothesize that NF‐κB signaling functionally regulates the BDNF/TrkB cascade via effects on microglial polarization states [82]. Thus, targeting microglial regulatory signaling pathways represents a promising strategy for MDD treatment.

## 3. Neuroinflammation and miRNAs in MDD

### 3.1. Mechanisms of miRNAs in Regulating Neuroinflammation in MDD

MiRNAs play a significant role in MDD pathogenesis by diverse mechanisms influencing modulating neuroinflammation. First, miRNAs can potentiate or attenuate neuroinflammatory responses via targeted gene regulation. Wu et al. [33] identified significantly reduced *miR-144-5p* expression in the hippocampal dentate gyrus of CUMS mice. Moreover, they found that *miR-144-5p* knockout in naive mice induced depressive‐like behaviors concurrent with neuronal abnormalities, including neuroinflammation, mediated by dysregulation of its target genes phosphatase and tensin homolog deleted on chromosome 10 and TLR4. He et al. [83] and Zhang et al. [84] reported that inhibition of *miR-142-5p* and *miR-155* could attenuates depressive symptomatology in CUMS mice and patients with depression, respectively, via modulation of neuroinflammation through BDNF targeting.

Second, miRNAs mediate intercellular communication via protein interactions to regulate extracellular neuroinflammation signaling. Through sequencing analysis, Xian et al. [34] found significantly elevated *miR-9-5p* expression in serum exosomes from patients with MDD compared to healthy controls. Neuronal overexpression of *miR-9-5p* has also been shown to exacerbate depressive phenotypes in CUMS mice. Mechanistically, *miR-9-5p* is transferred via neuron‐derived exosomes to microglia, promoting M1 polarization and increased release of proinflammatory cytokine (IL‐1*β*, IL‐6, and TNF‐*α*), thereby exacerbating neuroinflammation and neuronal damage.

Third, miRNAs may indirectly regulate neuroinflammation by modulating BBB integrity, HPA axis function, and neuroplasticity. Schell et al. [85] proposed that *miR-218* influences HPA axis regulation, neuronal development, and synaptic plasticity, impacting neuroinflammatory process in stress‐induced psychiatric disorders including MDD. Yang et al. [86] reported that *miR-542-3p* overexpression activates the Akt/GSK3*β*/*β*‐catenin pathway, alleviating corticosterone‐induced hippocampal neuronal damage in rodent depression models. Notably, *β*‐catenin exerts neuroprotective effects partially by preserving BBB integrity to suppress neuroinflammation [29].

Finally, miRNAs directly regulate key neuroinflammatory signaling pathways. For example, *miR-124*, *miR-130b-5p*, *miR-147a*, and *miR-223* have been shown to inhibit NF‐κB signaling [55, 58, 87, 88]. Conversely, the BDNF/TrkB pathway is suppressed by *miR-43b-5p*, *miR-134*, *miR-199a-5p*, and *miR-470-5p*, but activated by *miR-223* [55, 89–92].

### 3.2. The Potential of miRNAs as Therapeutic Targets for MDD

Given the regulatory role of miRNAs in neuroinflammation during MDD, targeting these molecules represents a promising therapeutic strategy. Several antidepressants and compounds with antidepressant potential show miRNAs‐specific activity. For example, the antihyperglycemic agent empagliflozin and the natural product ginsenoside Rb1 both exert antidepressant effects via targeted modulation of *miR-134* and downstream neuroinflammatory pathways, as previously described [28, 89]. Alzuri et al. [93] reported hippocampal downregulation of *miR-124-3p* and *miR-133b* in CUMS rats, with imipramine treatment reversing these alterations [32]. Functionally, *miR-124-3p* suppresses neuroinflammation by inhibiting IL‐1*β* release [94], while *miR-133b* restores dopaminergic neuronal function and mitigates neural damage [95], and both have been shown to ameliorate depressive‐like behaviors in rodent models. Notably, *miR-184-3p* is a common target of the selective serotonin reuptake inhibitors (SSRIs) fluoxetine and paroxetine. Mechanistic studies indicate that *miR-184-3p* negatively regulates immune‐related target genes, potentially delaying pathological immune pathway activation, attenuating excessive inflammation, and alleviating depression symptoms in CSDS mice [96, 97].

Beyond pharmacologically induced miRNAs modulation, direct administration of antagomirs could be used to suppress endogenous miRNAs activity, while direct administration of miRNAs mimics could restore deficient expression [30]. Preclinical evidence indicates that inhibition of *miR-142-5p* expression or overexpression of *miR-124*/*miR-133b* can reduce proinflammatory cytokine secretion and mitigate depressive symptomatology in CUMS rodents [83, 93]. As described earlier, Yang et al. [86] reported antidepressant and antineuroinflammatory effects using a neutralizing inhibitor to suppress *miR-542-3p*. Zheng et al. [98] employed siRNA targeting *miR-182-5p*, while Wang et al. [99] utilized circRNA protein tyrosine kinase 2 as a sponge for *miR-182-5p*, with both strategies alleviating neuroinflammation and depressive behaviors in CSDS and CUMS mice via *miR-182-5p* suppression. Luo et al. [100] engineered a novel DNA tetrahedral nanostructure for *miR-22-3p* delivery and reported that this nanocomplex improved depressive phenotypes and reduced IL‐1*β* expression in LPS‐induced depression models.

Despite compelling evidence supporting miRNAs as therapeutic targets for MDD, several challenges remain:

1. Translational validation is required to determine whether miRNAs targets identified in preclinical models have analogous regulatory functions in humans.
2. Controlled clinical studies are needed to establish if observed miRNAs dysregulation represents a core pathogenic feature of MDD or a treatment‐mediated epiphenomenon.
3. Practical implementation of such methods requires careful assessment of requisite resources, funding, and technical expertise [101].
4. Pharmacoepigenetic analyses, such as that by Prodan‐Barbulescu et al. [2], suggest differential miRNAs modulation by antidepressant classes: both SSRIs and serotonin‐norepinephrine reuptake inhibitors have been shown to significantly elevate *miR-212* levels in patients with MDD, but only SSRIs have been shown to increase *miR-183* and *miR-16*.
5. Lan et al. [102] and Guan et al. [103] reported diametrically opposite roles of *miR-204-5p*: downregulation promoted oxidative stress, neuroinflammation, and neuronal damage in CUMS rats, whereas upregulation suppressed neurogenesis in CSDS mice. Such findings suggest that miRNAs functions may be context‐dependent, with divergent or even opposing effects across different depression etiologies or species.

### 3.3. MiRNAs as Potential Biomarkers for MDD

As present, recognized biomarkers of MDD include CRP, BDNF, dopamine, and serotonin [104, 105]. However, measurement of dopamine and serotonin poses clinical challenges; peripheral concentrations correlate poorly with CNS levels, necessitating invasive cerebrospinal fluid sampling or costly positron emission tomography for accurate quantification [106]. Additionally, these neurotransmitters exhibit considerable instability and significant interindividual variability [104]. While CRP and BDNF are more accessible for extraction and detection [104], CRP reflects generalized systemic inflammatory without specificity for depression [107], and BDNF, although associated with neuroplasticity, lacks specificity across psychiatric disorders [108].

MiRNAs circulate in diverse biofluids including whole blood, serum, and cerebrospinal fluid. In psychiatric disorders, miRNAs are actively secreted or passively released from neurons into these compartments, frequently encapsulated within exosomes or microvesicles [109, 110]. Compared to traditional biomarkers, miRNAs offer superior sensitivity as potential MDD biomarkers and may be useful for guiding antidepressant selection and prognostic evaluation [32]. Funatsuki et al. [111] identified 141 differentially expressed miRNAs significantly associated with therapeutic response after 4 weeks of mirtazapine treatment in 46 patients. Class‐specific miRNA modulation by antidepressants, as discussed earlier [2], further supports their potential for treatment personalization. In a study of 64 patients with MDD, Kaurani et al. [112] observed significant downregulation of *miR-223-3p* in electroconvulsive therapy responders, with *miR-223-3p* expression positively correlating with proinflammatory cytokines (IL‐1*β*, IL‐6, and TNF‐*α*).

There is also evidence that miRNAs may be useful for differential diagnosis. For example, MDD and bipolar disorder type II (BD‐II) exhibit symptom overlap and can be clinically challenging to differentiate. Ni et al. [113] reported reduced peripheral blood *miR-16-5p* expression in patients with MDD versus healthy controls, with further reduction in unmedicated patients with BD‐II, suggesting its utility in distinguishing unmedicated BD‐II from MDD. Valiuliene et al. [114] reported significant correlation between *miR-16-5p* dynamics and antidepressant treatment response. Furthermore, *miR-93-5p* and *miR-146a-5p* exhibit predictive value for treatment response, which may relate to neuroinflammatory modulation [114].

However, there are key limitations regarding the use of miRNAs as biomarkers for MDD:

1. Many putative miRNAs biomarkers for MDD lack diagnostic specificity, with several failing to distinguish depression from BD or schizophrenia, constraining their diagnostic utility [11].
2. Certain CNS‐enriched miRNAs (e.g., *miR-124*) exhibit substantially lower peripheral concentrations, limiting feasibility as biomarkers due to the need for invasive sampling procedures [93].
3. The majority of research is confined to preclinical models, necessitating clinical validation.
4. There may be pathophysiological and miRNAs expression differences between geriatric and younger populations with MDD [115]. Consequently, combinatorial detection of multiple miRNAs signatures may represent a more specific diagnostic approach [32].

## 4. Natural Antidepressants Targeting miRNAs to Regulate Neuroinflammation

There is growing evidence that conventional antidepressants exert therapeutic effects through miRNAs‐mediated neuroinflammatory regulation. For example, fluoxetine and paroxetine require *miR-184-3p*‐dependent neuroinflammatory modulation for their antidepressant efficacy [96, 97], venlafaxine has been shown to ameliorate depressive‐like behaviors in CSDS mice through *miR-204-5p* downregulation [103], and imipramine and ketamine alleviate depressive phenotypes in CSDS models by normalizing dysregulated *miR-144-3p* expression to mitigate neuroinflammation [116]. However, these traditional pharmacotherapies have numerous limitations including frequent adverse effects [117, 118], development of pharmacotherapeutic resistance [119], and significant costs [120]. Natural medicinal compounds present an alternative with distinct therapeutic advantages such as favorable safety profiles, cost‐effectiveness, favorable pharmacokinetic properties, and structural diversity. Recent advances in molecular biology have elucidated the mechanistic basis of natural medicines’ efficacy for MDD management [121]. Here, we describe five structural categories of natural compounds showing significant therapeutic potential for MDD: saponins, flavonoids, alkaloids, polyphenols, and terpenoids (Table 1).

**Table 1:** Natural antidepressants targeting miRNAs to regulate neuroinflammation.

Categories	Natural antidepressants	Structure	Sources	Related miRNAs and signaling pathways	Experimental model	References
Saponins	Ginsenoside Rb1	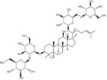	*Panax ginseng*	miRNAs: *miR-130b-5p* and *miR-134*. Signaling pathways: TLR4/NF‐κB.	In vitro: astrocytes and HAPI. In vivo: mice and rats.	[87, 89, 122]
	Notoginsenoside R1	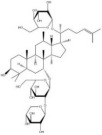	*Panax notoginseng*	miRNAs: *miR-29a* and *miR-147a*. Signaling pathways: PI3K/Akt/NF‐κB, MyD88/TRAF6/NF‐κB, and MAPK/Akt.	In vitro: H9C2 and HUVECs. In vivo: mice and rats.	[88, 123–126]
	Escin	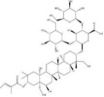	*Aesculus chinensis* and *Aesculus wilsonii*	miRNAs: *miR-223*. Signaling pathways: BDNF/TrkB/CREB and TLR4/MyD88/NF‐κB.	In vivo: rats.	[55, 127]

Saponins	Crocin	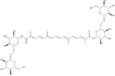	*Crocus sativus*	miRNAs: *miR-21*. Signaling pathways: TLR4/PTEN/Akt/mTOR/NF‐κB.	In vitro: HUVECs. In vivo: mice and rats.	[128–131]

Flavonoids	Liquiritigenin	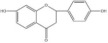	*Glycyrrhiza uralensis*	miRNAs: *miR-122-5p*. Signaling pathways: TLR4/MyD88/NF‐κB.	In vitro: hepatocytes. In vivo: mice.	[132–134]
	Baicalin	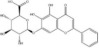	*Scutellaria baicalensis*	miRNAs: *miR-9-5p* and *miR-155*. Signaling pathways: TLR4/MyD88/NF‐κB/CREB and MAPK/ERK.	In vitro: BV‐2. In vivo: mice.	[40, 135–137]

Flavonoids	Oroxylin A	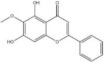	*Scutellariae radix*	miRNAs: *miR-182-5p* and *miR-183-5p*. Signaling pathways: BDNF/TrkB and CDK9/p53/VDAC.	In vitro: HEK293 and HT22. In vivo: mice.	[138, 139]
	Apigenin	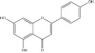	Various fruits and vegetables	miRNAs: *miR-15a*. Signaling pathways: CREB/BDNF.	In vivo: mice and rats.	[140, 141]

Alkaloids	Berberine	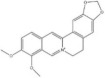	*Phellodendron chinense* and *Coptis chinensis*	miRNAs: *miR-34a*, *miR-43b-5p* and *miR-470-5p*. Signaling pathways: IKK*β*/NF‐κB, *miR-34b-5p*/*miR-470-5p*/BDNF and *miR-34a*/Bcl‐2.	In vitro: hippocampal neurons. In vivo: mice.	[142–144]

Alkaloids	Schisandrin B	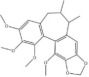	*Schisandra chinensis*	miRNAs: *miR-124*. Signaling pathways: TLR4/MyD88/NF‐κB.	In vitro: BV‐2.	[58, 145]

Polyphenols	Resveratrol	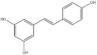	*Veratrum album* and *Polygonum cuspidatum*	miRNAs: *miR-134* and *miR-199a-5p*. Signaling pathways: NF‐κB/CREB/BDNF and SIRT1/eNOS.	In vitro: hippocampal neurons and HUVECs. In vivo: mice and rats.	[40, 90, 146, 147]
	Paenol	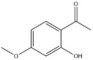	*Paeonis albiflora* and *Moutan cortex*	miRNAs: *miR-15a*. Signaling pathways: HDAC/*miR-15a* and HIF‐1*α*.	In vitro: N9. In vivo: mice and rats.	[148, 149]
Polyphenols	Curcumin	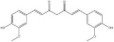	*Curcuma longa*	miRNAs: *miR-146a-5p* and *miR-199b-5p*. Signaling pathways: *miR-146a-5p*/ERK and *miR-199b-5p*/IKK*β*/NF‐κB/CREB.	In vitro: microglia. In vivo: rats.	[40, 150–152]

Terpenoids	Geniposide	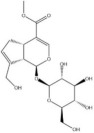	*Gardenia jasminoides*	miRNAs: *miR-25-3p*, *miR-298-5p* and *miR-511-3p* Signaling pathways: *miR-298-5p*/Nox1, *miR-25-3p*/Gata2, *miR-511-3p*/Fezf1/Akt, and Nrf2/ARE.	In vitro: frontal cortex neurons, HEK293T, hippocampal neurons, microglia, and RAW 264.7. In vivo: mice.	[153–156]

Terpenoids	Andrographolide	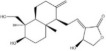	*Andrographis paniculata*	miRNAs: *miR-9-5p*, *miR-124-3p* and *miR-155* signaling pathways: TLR4/MyD88/NF‐κB and NLRP3.	In vitro: neurons. In vivo: mice and zebrafish.	[157–159]

*Note:* The chemical structures of the natural compounds were drawn using ChemDraw 18.0. BV‐2, BV‐2 murine microglial cell line; H9C2, H9C2 rat cardiomyoblast cell line; N9, murine microglial cell line N9; RAW 264.7, mouse leukemic monocyte macrophage cell line RAW 264.7; HT22, hippocampal neuron/HT22 cell line.

Abbreviations: Akt, protein kinase B; ARE, antioxidant response element; Bcl‐2, B‐cell lymphoma 2; BDNF, brain‐derived neurotrophic factor; CDK9, cyclin‐dependent kinase 9; CREB, cyclic adenosine monophosphate‐response element binding protein; eNOS, endothelial nitric oxide synthase; ERK, extracellular signal‐regulated kinase; Fezf1, fasciculation and elongation protein zeta family zinc finger 1; Gata2, GATA‐binding protein 2; HAPI, highly aggressively proliferating immortalized microglia; HDAC, histone deacetylase; HEK293, human embryonic kidney 293 cells; HEK293T, human embryonic kidney 293 cells, expressing SV40 large T antigen; HIF‐1*α*, hypoxia‐inducible factor 1, alpha subunit; HUVECs, human umbilical vein endothelial cells; IKK*β*, inhibitor of nuclear factor kappa‐B kinase subunit beta; MAPKs, mitogen‐activated protein kinases; miRNAs, micro ribonucleic acids; MyD88, myeloid differentiation primary response gene 88; NF‐κB, nuclear factor kappa‐B; NLRP3, nucleotide‐binding oligomerization domain‐like receptor pyrin domain containing 3; Nox1, nicotinamide adenine dinucleotide phosphate oxidase 1; Nrf1, nuclear factor erythroid 2‐related factor 1; p53, tumor protein p53; PI3K, phosphatidylinositol 3‐kinase; PTEN, phosphatase and tensin homolog deleted on chromosome ten; SIRT1, silent information regulator 2 homolog 1; TLR4, Toll‐like receptor 4; TRAF6, tumor necrosis factor receptor‐associated factor 6; TrkB, tropomyosin receptor kinase B; VDAC, voltage‐dependent anion channel.

### 4.1. Saponins

Ginsenoside Rb1 is the primary bioactive component of *Panax ginseng*. Li et al. [122] established that ginsenoside Rb1 inhibits LPS‐induced inflammation and pyroptosis in cultured astrocytes. Ginsenoside Rb1 has been shown to activate astrocytic autophagy in CUMS rats, suppressing inflammatory responses and pyroptotic cell death, attenuating synaptic structure damage, and ameliorating depressive‐like behaviors. Wang et al. [87] described ginsenoside Rb1‐mediated attenuation of microglial inflammation and neuronal damage through upregulation of *miR-130b-5p* expression, a pathway implicated in MDD symptom relief [55]. A complementary study by Wang et al. [89] suggested that ginsenoside Rb1 improves depressive phenotypes in CUMS mice by downregulating *miR-134* expression to enhance hippocampal synaptic plasticity.

Notoginsenoside R1, which is derived from *Panax notoginseng*, exhibits antiapoptotic, antioxidant, and anti‐inflammatory properties [123]. Findings by Zhan et al. [123] suggest the antidepressant efficacy of notoginsenoside R1 in CUMS rats through suppression of neuroinflammation and hippocampal neuronal apoptosis resulting from PI3K/Akt/NF‐κB pathway activation. Wang et al. [124] found that notoginsenoside R1 counteracts anesthetic‐induced hippocampal inflammation, exerting neuroprotective effects, which are abolished by *miR-29a* knockdown. A clinical study by Maffioletti et al. [160] found that reduced *miR-29a* expression was associated with poor response to trauma‐focused psychotherapy in patients with MDD. Li et al. [88] established that notoginsenoside R1 upregulates *miR-147a* expression, which concurrently inhibits the MyD88/TRAF6/NF‐κB and Akt/mammalian target of rapamycin (mTOR) signaling pathways (described in Sections 2.2 and 3.1) [161] to mitigate oxidative stress and neuroinflammation. Additional studies indicate that the anti‐inflammatory actions of notoginsenoside R1 depend on suppression of NF‐κB and MAPK cascades [125, 126]. These mechanisms, together with the aforementioned studies [66, 123], support the potential therapeutic potential of notoginsenoside R1 for depression.

Escin, the active component of *Aesculus chinensis* and *Aesculus wilsonii* seeds (traditional Chinese medicine “Suo Luo Zi”), possesses established neuroprotective and anti‐inflammatory activities [127]. Liu et al. [55] found that escin significantly ameliorates depressive symptoms and neural damage in CUMS rats, with its efficacy comparable to that of fluoxetine. Mechanistically, escin activates hippocampal *miR-223* expression, resulting in inhibition of the NF‐κB pathway but enhancement of BDNF/TrkB pathway activity, thereby reducing neuroinflammation and counteracting depression‐associated neuropathology [29, 55].

Crocin, a principal bioactive component of *Crocus sativus*, exhibits antidepressant and anticancer properties through anti‐inflammatory and antioxidant mechanisms [128]. Abbaszade‐Cheragheali et al. [128] reported that crocin ameliorates depressive behaviors in CUMS rats by reducing cerebral oxidative stress, serum inflammatory cytokines, and corticosterone levels. Zununi Vahed et al. [129] identified crocin‐mediated suppression of inflammation and cytoprotection through *miR-21* downregulation. Joodaki et al. [130] reported superior antidepressant and anxiolytic efficacy with crocin‐escitalopram combination therapy compared to monotherapy in depression model rodents. A clinical study by Chen et al. [162] found that elevated *miR-21* expression in patients with MDD was associated with impaired neural connectivity and synaptic plasticity. Xiao et al. [131] established that gut microbiota metabolize oral crocin into active crocetin, with dysbiosis impairing this biotransformation and diminishing antidepressant efficacy, indicating that microbial conversion is a key determinant of crocin’s therapeutic activity.

### 4.2. Flavonoids

Liquiritigenin, a dihydroflavonoid compound derived from *Glycyrrhiza uralensis* roots, exhibits potent antioxidant and anti‐inflammatory properties that may underlie its neuroprotective effects in neurological disorders [121, 132]. Shen et al. [133] reported that liquiritigenin ameliorates depressive behaviors through NF‐κB pathway inhibition. A study by Sharma et al. [132] corroborates these neuroinflammatory‐modulating and antidepressant properties. Regarding the mechanism, there is evidence that liquiritigenin targets *miR-122-5p*. For example, Yang et al. [134] observed increased hepatic *miR-122-5p* expression following liquiritigenin treatment in insulin‐resistant mice, which reversed metabolic dysfunction. A clinical study by Krivosova et al. [163] identified significantly reduced plasma *miR-122-5p* levels in patients with depression, with levels normalizing after antidepressant therapy. Wang et al. [164] established that *miR-122-5p* overexpression suppresses proinflammatory cytokine (IL‐1*β* and TNF‐*α*) release from microglia and astrocytes. However, direct evidence linking liquiritigenin’s antidepressant effects to *miR-122-5p* modulation is currently limited.

Baicalin, a bioactive flavonoid isolated from *Scutellaria baicalensis* roots, demonstrates diverse pharmacological activities [40, 165]. Yi et al. [135] employed integrative bioinformatics, including overlapping terms analysis, protein–protein interaction network topology, and pathway enrichment, to identify baicalin’s ability to concurrently target oxidative stress, apoptotic, and inflammatory pathways in depression. Baicalin has been shown to specifically modulate *miR-9-5p* and *miR-155*. *MiR-9-5p*, which is transported via neuron‐derived exosomes to microglia in MDD, promotes microglial M1 polarization and neurotoxicity [34], whereas elevated *miR-155* is correlated with greater MDD symptom severity [84]. The baicalin‐dominated formulation of the Xiaoxuming decoction has been shown to inhibit neuroinflammation in LPS‐induced microglia by suppressing *miR-9-5p* [136]. Baicalin also attenuates neuroinflammation in LPS‐induced microglia by blocking TLR4‐mediated signaling and *miR-155* expression [137].

Oroxylin A, a neuroprotective and anti‐inflammatory flavonoid from *Scutellariae radix*, has been shown to mitigate depressive behaviors in CUMS and CRS mice through BDNF/TrkB pathway activation [138]. Xia et al. [139] found that oroxylin A normalizes depression‐associated upregulation of *miR-182-5p* and *miR-183-5p* in neuronal tissue, ameliorating behavioral deficits. Functionally, increased levels of *miR-182-5p* drive neuroinflammation and depressive phenotypes in CUMS and CSDS models, whereas inhibition of *miR-182-5p* confers therapeutic benefits [98, 99]. *MiR-183-5p* overexpression has been observed in hippocampal and ventral tegmental areas of individuals with neuroinflammation‐associated conditions including depression [166, 167].

Apigenin, a widely distributed dietary flavonoid, also shows significant neuroprotective effects [140]. Olayinka et al. [140] reported that apigenin reverses hippocampal and prefrontal cortical neuronal loss by inhibiting neuroinflammation, ameliorating anhedonia in CUMS mice. Taha et al. [141] found that apigenin modulates microglial polarization states and attenuates neuroinflammation/oxidative stress through *miR-15a* regulation, suggesting *miR-15a* mediation of its antidepressant effects.

### 4.3. Alkaloids

Berberine, an isoquinoline alkaloid derived from *Phellodendron chinense* and *Coptis chinensis*, has established clinical applications in managing diarrhea. However, emerging evidence of its broader therapeutic potential, including applications in cardiovascular, neurological, and psychiatric disorders. The antidepressant activity of berberine is mediated by neuroinflammation suppression [142, 143]. Yi et al. [144] indicated that berberine reduces depressive behaviors in CUMS mice by enhancing dendritic spine morphology and hippocampal neurogenesis through *miR-34a* downregulation. Liu et al. [168] found that *miR-34a* exacerbates neuroinflammation, impairing neuronal function and viability. It is thought that *miR-43b-5p* and *miR-470-5p* contribute to depression pathogenesis via neuroinflammation induction and BDNF targeting [91]. Zhan et al. [143] reported berberine‐mediated attenuation of depressive symptoms in CUMS mice, concomitant with hippocampal neuron growth promotion through *miR-43b-5p* and *miR-470-5p* suppression. Berberine was also found to counteract miRNA‐mediated dysregulation via BDNF overexpression, normalizing hippocampal neuroplasticity and depressive behaviors in CUMS mice.

Schisandrin B, a bioactive lignan compound isolated from *Schisandra chinensis* fruit, shows potential for managing neuropsychiatric disorder [58, 145]. Using network pharmacology and molecular docking analysis, Mokhtari et al. [145] reported the antidepressant potential of schisandrin B through TNF pathway modulation. An in vitro study by Yang et al. [58] using primary microglial cultures found that schisandrin B upregulates *miR-124* expression and suppresses MAPK and NF‐κB/TLR4/MyD88 signaling cascades, thereby attenuating neuroinflammation and exerting antidepressant effects. These effects were abolished by *miR-124* inhibition, confirming pathway specificity.

### 4.4. Polyphenols

Resveratrol, a polyphenolic compound derived from *Veratrum album* and *Polygonum cuspidatum* [90], demonstrates antidepressant efficacy through neuroinflammatory modulation [40]. Shen et al. [90] established that resveratrol downregulates *miR-134* expression both in vitro and in vivo, ameliorating depressive behaviors and preventing comorbid cognitive deficits in CUMS rats. Complementary evidence implicates *miR-199a-5p* in depression pathogenesis through proinflammatory mechanisms [92, 169]. Liu et al. [92] found that *miR-199a-5p* exacerbates depression by inhibiting the cyclic adenosine monophosphate‐response element binding protein (CREB)/BDNF pathway, with its pharmacological inhibition attenuating depressive phenotypes in CUMS mice. Zhang et al. [146] identified resveratrol as a potential *miR-199a-5p* suppressor. Ibrahim et al. [147] reported synergistic attenuation of neuroinflammation and depressive‐like behaviors between resveratrol and 17*β*‐estradiol in CRS mice, warranting further investigation of its potential synergistic interactions with conventional antidepressants.

Paenol, a phenolic compound isolated from *Paeonis albiflora* and *Moutan cortex*, exhibits validated anti‐inflammatory and antioxidant properties [121, 148]. Cai et al. [148] reported paenol‐mediated neuroinflammatory suppression through *miR-15a* upregulation in rodent models. Zhang et al. [149] established that paenol reduces microglial proinflammatory cytokine (IL‐6 and TNF‐*α*) expression in LPS‐induced depression models via hypoxia‐inducible factor 1, alpha subunit (HIF‐1*α*) pathway modulation, significantly attenuating depressive behaviors. There is notable evidence that *miR-15a* overexpression ameliorates neuroinflammation through dual regulation of CREB/BDNF and HIF‐1*α* signaling [141, 170]. These findings collectively suggest that paenol exerts antidepressant effects via coordinated *miR-15a* upregulation and HIF‐1*α* pathway modulation, although the precise molecular mechanisms require further elucidation.

Curcumin, a natural bioactive polyphenol from the rhizomes of *Curcuma longa* and other *Zingiberaceae* species, has attracted interest for treating neuropsychiatric disorders due to its favorable gastrointestinal absorption and BBB permeability [40, 150, 151]. Nguyen et al. [150] found that *miR-146a-5p* exhibited the strongest binding affinity of 74 depression‐associated miRNAs targeted by curcumin, indicating its pivotal role in curcumin’s antineuroinflammation and antidepressant activity. A study by Fan et al. [151] corroborated this finding, showing that curcumin normalizes elevated hippocampal *miR-146a-5p* expression in LPS‐induced depressive rats, which reduced oxidative stress, neuroinflammation, and synaptic dysfunction, to confer neuroprotection. Another key target of curcumin is *miR-199b-5p*. Li QS et al. [171] found that reduced *miR-199b-5p* expression was associated with increased relapse risk in patients with MDD, Gao et al. [152] reported that curcumin upregulates *miR-199b-5p* in microglia and significantly attenuates inflammatory response in LPS‐activated cells. Crucially, *miR-199b-5p* knockdown abrogated the anti‐inflammatory effects of curcumin, suggesting that this miRNA mediates both the therapeutic effects of curcumin and neuroinflammation‐associated relapse risk in MDD.

### 4.5. Terpenoids

Geniposide, a natural iridoid glycoside purified from *Gardenia jasminoides* [153], has been shown to modulate neuroinflammation and exert antidepressant effects through miRNA‐mediated mechanisms [153–155]. Zhao et al. [154] reported that geniposide downregulates *miR-25-3p* and alters associated signaling networks, ameliorating depressive behaviors in CUMS mice. Zare‐Chahoki et al. [172] reported strong positive correlations between *miR-25-3p* and proinflammatory cytokine (IL‐1*β*, IL‐6, and TNF‐*α*) levels. Complementary studies by Zou et al. [153, 155] suggest that geniposide elevates *miR-298-5p* expression, thereby suppressing its target nicotinamide adenine dinucleotide phosphate oxidase 1 and attenuating neuroinflammation in CUMS models. Geniposide may also reduce neuronal oxidative stress and alleviate depressive symptomatology through *miR-511-3p* suppression. A nanotherapeutic advancement devised an exosome‐functionalized geniposide‐loaded Prussian blue delivery system reported significantly enhanced BBB penetration efficiency and superior efficacy for modulating microglial polarization, attenuating neuroinflammation, and conferring antidepressant activity [156].

Andrographolide, a diterpenoid lactone from *Andrographis paniculata*, exhibits antiviral, antitumor, antifibrotic, and anti‐inflammatory properties [165] and has been shown to ameliorate depressive‐like behaviors in CUMS zebrafish [157]. Mechanistically, andrographolide exerts antidepressant effects through two pathways: suppression of nucleotide‐binding oligomerization domain‐like receptor pyrin domain containing 3 inflammasome activity and enhancement of autophagic flux [157, 158]. Notably, these effects were attenuated by the autophagy inhibitor chloroquine. Studies have identified *miR-9-5p*, *miR-124-3p*, and *miR-155* as potential mediators of andrographolide’s regulation of autophagy and neuroinflammation [159], which have been previously implicated in depression pathogenesis [34, 58].

## 5. Discussion and Future Directions

As the most prevalent mental health condition, MDD is a significant focus of psychology and neurobiology research. MDD imposes considerable medical burden on affected individuals and their families, as well as substantial costs for governments due to its associated social problems. Due to current incomplete understanding of MDD etiology, existing treatment options are suboptimal or ineffective.

Although the pathogenesis of MDD is highly complex, neuroinflammation is now recognized as a key contributor. Patients with inflammatory diseases demonstrate increased susceptibility to mood disorders, and depression increases vulnerability to severe physical illnesses [4]. However, a definitive causal link between neuroinflammation and depression is lacking. Haider et al. [173] reported that patients with immune‐mediated inflammatory diseases (e.g., rheumatoid arthritis and inflammatory bowel disease) exhibit a higher depression of prevalence, have higher healthcare costs, and poorer treatment compliance, underscoring the need for clinical monitoring of these patients to identify early depression symptoms and provide timely intervention. Similarly, Li et al. [174] reported that comorbid MDD in type 2 diabetes mellitus correlates with higher complication rates, which may relate to inflammation‐associated circulating metabolites. These findings suggest a bidirectional relationship between neuroinflammation and depression. As neuroinflammation is intrinsically linked to various biological processes, including autophagy, programmed cell death, and oxidative stress, future research must integrate these elements to achieve a comprehensive understanding of neuroinflammation’s role in depression [5, 41, 42, 135, 175]. Investigation of this association could facilitate the development of novel antidepressants.

As detailed in this review, there is growing evidence that miRNAs are molecular bridges linking neuroinflammation and depression. Targeting miRNAs to modulate neuroinflammation as part of treatment for MDD and utilizing miRNAs for treatment selection and prognosis assessment have become important research directions with substantial empirical support. However, there are significant challenges before these findings can result in clinical translation.

1. There may be discordance between plasma and brain tissue miRNA profiles. Obtaining brain tissue samples for miRNAs analysis remains impractical in clinical studies.
2. MiRNAs composition differs significantly between animal models and humans, with expression patterns and functions exhibiting interspecies variation. Even within human populations, there is evidence of age‐ and sex‐dependent miRNAs expression variations [176]. Due to the vast number of miRNA species, repeatedly validated molecules should be prioritized for further research (Table 2).
3. Therapeutic strategies directly targeting specific miRNAs are hindered by the clinical unsuitability of existing delivery systems (e.g., lentiviral injections and siRNA). Long‐term miRNAs modulation risks off‐target effects and adverse reactions due to unintended target interactions [177]. Consequently, structural modification or optimizing the formulation of established antidepressants with confirmed miRNAs‐regulating activity may be a more feasible approach [40, 100, 131, 156].
4. Expression changes of individual miRNAs vary across animal models of depression induced by different methodologies, suggesting that depression subtypes may have divergent miRNA signatures [102, 103]. Furthermore, miRNA effects can differ or oppose each other depending on cellular microenvironments, complicating outcome prediction for clinical interventions [177]. Moreover, certain miRNAs demonstrate similar expression profiles across multiple psychiatric disorders (e.g., MDD, schizophrenia, and BD) [178], compromising their specificity as biomarkers.
5. Clinical implementation of miRNA biomarkers necessitates the establishment of validated, standardized reference ranges.

Despite the availability of diverse antidepressants (e.g., sertraline, fluoxetine, and venlafaxine), many patients with MDD experience adverse effects and treatment resistance. Natural medicines have received increasing research attention due to their lower toxicity profiles and availability. Numerous natural medicines demonstrate antidepressant activity, with some exerting effects via miRNAs‐mediated neuroinflammatory regulation. These agents thus hold promise as alternative treatments for MDD. However, future research on the therapeutic applications for MDD will need to address the following points:

**Table 2:** Key miRNAs regulating neuroinflammation and MDD pathological processes.

miRNAs	Regulatory orientation in MDD	Target molecules	Pathological mechanisms associated with neuroinflammation	Interventional drugs and therapies	References
*miR-9-5p*	↑	MAPK and NF‐κB	Promoting M1 polarization of microglia and regulate autophagy	Baicalin and andrographolide	[34, 136, 159]
*miR-15a*	↓	BDNF and CREB	Promoting M2 polarization of microglia and attenuate oxidative stress	Apigenin and paenol	[141, 148, 170]
*miR-16-5p*	↓	BDNF	Enhance neuroplasticity	rTMS	[113, 114]
*miR-34a*	↑	Bcl‐2 and CREB	Impair neurogenesis	Berberine	[144, 168]
*miR-43b-5p*	↑	BDNF	Impair neuroplasticity	Berberine	[91, 143]
*miR-124*	↓	MAPK and NF‐κB	Reduce ROS production, promoting M2 polarization of microglia, and regulate autophagy	Schisandrin B and andrographolide	[32, 58, 93, 94, 159]
*miR-134*	↑	BDNF and NF‐κB	Impair neuroplasticity	Empagliflozin, ginsenoside Rb1 and resveratrol	[28, 89, 90]
*miR-144*	↑	MAPK and NF‐κB	Promoting M1 polarization of microglia, impair neurogenesis, and neuroplasticity	Imipramine and ketamine	[33, 116]
*miR-146a-5p*	↑	ERK	Induce oxidative stress and synaptic dysregulation	Curcumin	[114, 150, 151]
*miR-147a*	↓	MAPK and NF‐κB	Promoting M2 polarization of microglia and mitigating oxidative stress	Notoginsenoside R1	[88, 161]
*miR-155*	↑	MAPK and NF‐κB	Promoting M2 polarization of microglia and regulate autophagy	Fluoxetine hydrochloride, Baicalin, and andrographolide	[84, 137, 159]
*miR-182-5p*	↑	CDK9	Disrupting HPA axis homeostasis	Oroxylin A	[98, 99, 139]
*miR-199a-5p*	↑	BDNF	Impair neuroplasticity	Resveratrol	[92, 146, 169]
*miR-204-5p*	↓ (CUMS)	RGS12	Alleviate oxidative stress and neuronal damage	Flutamide and corticosterone	[102]
	↑ (CSDS)	BDNF	Impair neuroplasticity and neurogenesis	Venlafaxine	[103]
*miR-223*	↓	BDNF and NF‐κB	Enhance neuroplasticity and reduce ROS production	Escin and fluoxetine	[55, 112]
*miR-470-5p*	↑	BDNF	Impair neuroplasticity	Berberine	[91, 143]

*Note:* In the “Regulatory orientation in MDD” column, “↑” indicates upregulation of the miRNA in MDD, while “↓” represents downregulation. Notably, *miR-204-5p* is downregulated in the CUMS model but upregulated in the CSDS model [102, 103].

Abbreviations: Bcl‐2, B‐cell lymphoma 2; BDNF, brain‐derived neurotrophic factor; CDK9, cyclin‐dependent kinase 9; CREB, cyclic adenosine monophosphate‐response element binding protein; CSDS, chronic social defeat stress; CUMS, chronic unpredictable mild stress; ERK, extracellular signal‐regulated kinase; HPA, hypothalamic–pituitary–adrenal; MAPKs, mitogen‐activated protein kinases; MDD, major depressive disorder; miRNAs, micro ribonucleic acids; NF‐κB, nuclear factor kappa‐B; RGS12, regulator of G protein signaling 12; ROS, reactive oxygen species; rTMS, repetitive transcranial magnetic stimulation.

1. As MDD arises from multigenic dysregulation, elucidation of how natural medicines modulate miRNA networks within antidepressant mechanisms is essential.
2. Natural medicines typically target multiple miRNAs; However, synergistic interactions between these targets and their relative contributions require further characterization.
3. Research should focus on modifying the chemical structures or formulations of validated natural medicines to enhance their bioavailability and efficacy [40, 131, 156]. Synergistic effects observed between certain phytomedicines and conventional antidepressants [130, 147] warrant consideration in clinical treatment planning.
4. While generally safe, natural medicines can exert nonspecific off‐target effects and have associated toxicities. Documented adverse reactions to natural medicine include embryotoxicity and teratogenicity to ginsenoside Rb1 [179]; Hypersensitivity and potential seizure induction (with bupropion coadministration) for baicalin and geniposide [180, 181]; Gastrointestinal toxicity, hepatotoxicity, and arrhythmias with berberine [181, 182]; Arthralgia and thrombocytopenia with schisandrin B [181]; Increased cardiovascular disease risk in elderly individuals, hormonal dysregulation, and gastrointestinal effects with resveratrol [183], and nausea/diarrhea with curcumin [184]. Clinicians should monitor these effects, and research efforts should prioritize modifications to mitigate toxicity.
5. Rigorous randomized double‐blind clinical trials are needed to establish the efficacy, long‐term outcomes, synergistic effects, adverse event profiles, optimal dosing, and potential drug interactions of natural medicines showing antidepressant activity.

## 6. Conclusion

Greater understanding of miRNAs regulation of neuroinflammation in MDD is critical for elucidating the pathogenesis of this mental health condition and informing personalized treatment. Despite growing research on this topic, notable limitations exist:

1. Most mechanistic insights have been derived from rodent models of depression, and robust clinical corroboration is lacking.
2. Challenges persist in developing effective delivery systems for direct miRNAs modulation. Furthermore, the roles of miRNAs in depression are complex and heterogeneous (e.g., *miR-204-5p* shows opposing expression patterns in different depression models [102, 103]) and lack specificity (common alterations across different psychiatric disorders [178]). These factors impede miRNAs research and limit their utility as biomarkers. Additionally, standardized clinical reference ranges for miRNA biomarkers are lacking.
3. Further research on natural medicines with antidepressant effects is needed to elucidate their mechanisms (especially the target synergy) and rigorously validate clinical efficacy, safety, and bioavailability through clinical trials to accelerate translation.

## Conflicts of Interest

The author declares no conflicts of interest.

## Author Contributions

**Qirui Li:** investigation, writing – original draft, writing – review and editing, visualization. **Yuyan Ling**: writing – review and editing, visualization. **Ling Gu**: writing – review and editing, visualization. **Lei Li, Yutong Liu, and Yuanyuan Ma**: writing – review and editing. **Ruiting Ma**: funding acquisition. **Meijuan Chen**: conceptualization, funding acquisition.

## Funding

This work was supported by the Clinical Need Oriented Basic Research Project of the Inner Mongolia Academy of Medical Sciences (Grant 2023GLLH0154) and the Scientific Research Project of the Inner Mongolia Autonomous Region Mental Health Center (Grant 2022MSWN001).

## Data Availability

The data sharing is not applicable to this article as no datasets were generated or analyzed during the current study.
